# Culture negative empyema in a critically ill child: an opportunity for rapid molecular diagnostics

**DOI:** 10.1186/1471-2253-14-107

**Published:** 2014-11-22

**Authors:** Elsa L Vazquez Melendez, John J Farrell, Andrea M Hujer, Kristin S Lowery, Rangarajan Sampath, Robert A Bonomo

**Affiliations:** Departments of Medicine & Pediatrics, University of Illinois School of Medicine, 530 N.E. Glen Oak Ave, Peoria, IL 61603 USA; Department of Medicine, University of Illinois School of Medicine, Peoria, IL USA; Louis Stokes Cleveland Department of Veterans Affairs Medical Center, Research Service, 10701 East Blvd, Cleveland, Ohio 44106 USA; Department of Medicine, Case Western Reserve University, Cleveland, OH USA; Ibis Biosciences, an Abbott Company, Carlsbad, CA USA; Departments of Pharmacology and Molecular Biology and Microbiology, Case Western Reserve University, Cleveland, OH USA

**Keywords:** Empyema, Culture negative infection, Antimicrobial stewardship, Pediatric infection

## Abstract

**Background:**

Nucleic acid amplification technologies (NAAT) are advancing our ability to make rapid molecular diagnoses in patients with serious culture negative infections. This is the first report of PCR coupled to electrospray ionization mass spectrometry use in the evaluation of complicated community acquired pneumonia in a pediatric patient.

**Case presentation:**

We present a case of culture negative empyema in a critically ill, Caucasian, 2-year-old girl who was treated with broad-spectrum empiric antibiotics, in which the length of stay was prolonged by adverse effects of the empiric antibiotic treatment. PCR coupled to electrospray ionization mass spectrometry was applied to culture negative fluid and tissue samples from the patient in order to determine the etiology of the empyema.

**Conclusions:**

Using this method, *Streptococcus mitis/viridans* was identified as the pathogen. A retrospective review of cases of empyema in children at our institution found that 87.5% of cases were negative for identification of a pathogen and antibiotics were administered to 100% of cases prior to collecting pleural fluid for culture. Understanding the role of *Streptococcus mitis/viridans* group in the etiology of empyema using an advanced NAAT coupled with mass spectrometry can enlighten clinicians as to the impact of this pathogen in community acquired pneumonia and help assist with antibiotic stewardship.

## Background

Despite the introduction of the pneumococcal conjugate vaccine into the childhood immunization schedule in 2000, community acquired pneumonia (CAP) remains the most common infectious cause for hospitalization in children. Given the impact of the pneumococcal vaccine on pneumonia incidence, more uncertainty exists today regarding the most likely microbiologic etiology when cultures are negative. Additionally, empyema is a common complication of pediatric CAP. In more than 60% of cases, pleural fluid or tissue fails to yield a pathogen. Therefore, selecting appropriate antimicrobial therapy for patients with evidence of infection, but negative cultures is a common dilemma in practice.

Herein, we describe the application of PCR followed by electrospray-ionization mass spectrometry (PCR/ESI-MS) for detection of *Streptococcus mitis* directly from “culture negative” pleural fluid in a 2 year-old girl with a loculated parapneumonic effusion that developed during broad spectrum empiric IV antibiotic treatment. This case illustrates that PCR/ESI-MS may have a role as an adjunct to conventional diagnostic microbiologic methods in cases when cultures of specimens obtained following the initiation of antibiotic treatment do not yield a pathogen.

## Case presentation

A previously healthy two-year-old girl was transferred to our tertiary care center from her pediatrician’s office in acute respiratory distress. Her symptoms began six days before arrival with nasal congestion, sore throat, non-productive cough and fever; her oral intake and activity level had both decreased, as well. Her two siblings had recently recovered from mild upper respiratory infections, presumed to be due to a viral infection. The patient also complained to her parents of pain in the ribs and abdomen. In her pediatrician’s office, point of care testing for Respiratory Syncytial Virus (RSV) and Group A β-hemolytic streptococcal (GAS) infection were both negative.

Her past medical history was unremarkable. She had been a full term infant who was born by vaginal delivery without complications. She had been diagnosed with RSV infection at eight months of age, and had an uneventful recovery. Her immunizations were up-to-date, and she had no allergies. She lives with mother, father and two siblings, all of whom are non-smokers. The family has no pets, and the family history was unremarkable. In the developmental history, all milestones had been achieved in a normal sequence and time.

On exam, she was initially afebrile: axillary temperature was 36.6°C, respiratory rate and heart rate were 20 breaths/min and 138 beats per minute, respectively and blood pressure was 138/77 mmHg. Height was 36.6″ inches (63% percentile) and weight 16.3 kg (96% percentile). Pulse oximetry was 93% on room air. She was listless. Her head, ear, eyes, nose and throat exam were unremarkable. There was no cervical lymphadenopathy. She was tachycardic, but the rhythm was regular, and no murmur was appreciated. Pulmonary examination was notable for diminished breath sounds on the left. Abdominal, neurological, skin and musculoskeletal exam were unremarkable.

**Figure 1:**
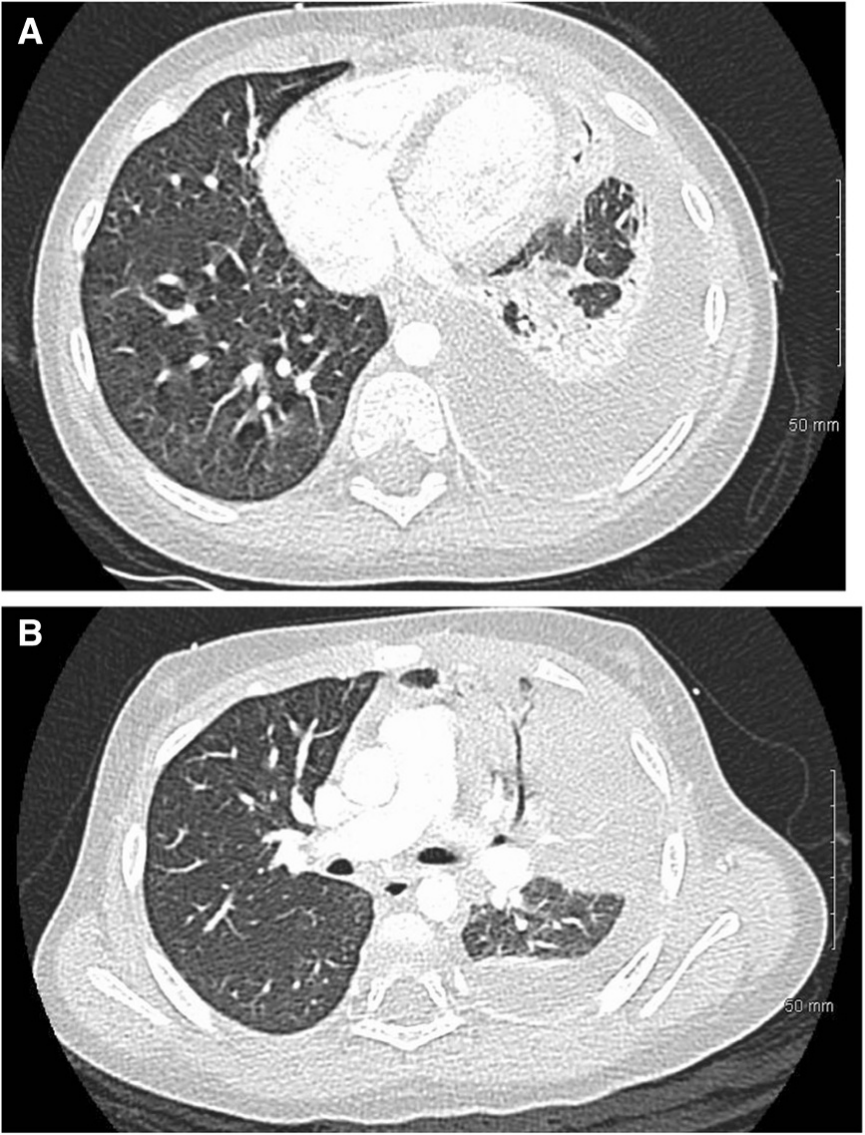
**Images of CT scan of chest obtained from 2 year old child with empyema. A)** Large pleural effusion with resulting left lower lobe compressive atelectasis. **B)** Left upper lobe consolidation with air bronchogram.

Laboratory studies on the day of admission showed leukocytosis, anemia, and decreased albumin (Table 1). A two view chest X-ray was notable for a left lung infiltrate. Low dose screening computer tomography (CT) scan of the chest demonstrated a left upper lobe pneumonia with decreased contrast enhancement of the left upper lobe, concerning for left upper lobe ischemia, and a moderate to large left pleural effusion, but no evidence for empyema or compressive atelectasis of the left lower lobe (Figure 1).

**Table 1:** **Laboratory results at admission, midpoint of hospital stay, and discharge**

Complete blood count	Day 1	Day 5	Day 10
White blood cell count	17,000 cells/dL	19,400 cells/dL	13,200 cells/dL
Differential	69% neutrophils	73% neutrophils	64% neutrophils
	18% lymphocytes	17% lymphocytes	27% lymphocytes
	13% monocytes	9% monocytes	8% monocytes
Hemoglobin	8.7 g/dL	7.3 g/dL	8.8 g/dL
Hematocrit	26%	23%	23%
Platelets	314 thous/UL	350 thous/UL	595 thous/UL
Mean corpuscular volume of (Hct/RBC count x 10)	74 femtoliters	79 femtoliters	79 femtoliters
**Comprehensive metabolic profile**			
Sodium	138 mmol/L	136 mmol/L	Not repeated
Potassium	3.9 mmol/L	4.7 mmol/L	
Chloride	106 mmol/L	108 mmol/L	
Bicarbonate	21 mmol/L	21 mmol/L	
Blood urea nitrogen	13 mg/d	2 mg/d	
Creatinine	0.51 mg/dl	0.36 mg/dl	
Total protein	5.6 gm/d	4.8 gm/d	
Albumin	2.7 gm/dl	2.1 gm/dl	
Calcium	8.8 mg/dl	8.5 mg/dl	
Total bilirubin	0.4 mg/dl	0.5 mg/dl	
Alanine transaminase	25 U/L	26 U/L	
Aspartate transaminase	22 U/L	21 U/L	
Alkaline phosphatase	186 U/L	114 U/L	
**Microbiology**	**Blood cultures**	**Pleural Fluid**	**Operative (VATS)**
BACTEC™ culture bottles	No growth	No growth	–
Aerobic Culture	–	–	No growth
PCR/ESI-MS	–	*mitis/viridans* streptococci	*mitis/viridans* streptococci

Following the initial evaluation, empiric intravenous (IV) antibiotics were started: IV clindamycin and ceftriaxone. Despite therapy, she continued to experience fevers to 39°C. On hospital day 2, blood cultures from admission remained negative, but because of persistent fever (T 39.4°C), tachycardia (HR 175), respiratory distress, and leukocytosis (WBC count = 17,000 cells/dL) a diagnostic thoracoscopy with placement of a 20 French chest tube (CT) into the posterior left pleural space was performed. During the thoracoscopy, a 5 mm camera was inserted to inspect the lung and pleural space, which was found to contain straw colored fluid that was free of fibrinous material. Pleural fluid was submitted for both aerobic and anaerobic cultures, which were negative. Gram stain showed no organisms.

The patient remained febrile with daily temperatures up to 40.0°C (104°F) and tachycardic (sinus HR ranged from 136 to 175 bpm). On hospital day five, cultures of the fluid drained during CT insertion remained negative (Table 1). A repeat ultrasound of the chest showed a complex left pleural effusion containing internal debris and septations. In spite of three tissue plasminogen activator infusions through the chest tube, the effusion remained loculated and failed to drain. On hospital day six, video-assisted thoracic surgery (VATS) with decortication of necrotic tissue was performed along with placement of two large bore chest tubes. Cultures of tissue obtained during the VATS procedure were negative. On hospital day eight, the patient developed abdominal distention secondary to adynamic ileus. Stool softeners were started which helped regulate stool patterns as well as the ileus. She received a total of 9 days of IV clindamycin and IV ceftriaxone. On the day of discharge, hospital day 10, the chest tubes were removed, and the antibiotic treatment was changed to oral amoxicillin/clavulanate. She tolerated this medication well, and she was discharged with a plan to complete 10 additional days of treatment (Table 1).

The patient was seen in follow-up two days following hospital discharge. Her mother reported that she was tolerating the oral antibiotic, she had no diarrhea and her appetite had improved, but she continued to have abdominal distension. She was afebrile. Her lung exam was still notable for diminished breath sounds at the left base. The abdomen was mildly distended; surgical incisions were intact and healing nicely and there was no evidence of active infection. Conservative measures were prescribed for treatment of the abdominal distension. She completed antimicrobial treatment without further incident, and recovered completely from her infection.

Informed consent was obtained from the parents of the patient for publication of this case report and any accompanying images. A copy of the written consent is available for review by the Editor of this journal.

### PCR/ESI-MS

PCR/ESI-MS is a rapid molecular diagnostic platform that identifies pathogens across the entire spectrum of disease processes: bacteria, viruses, parasites, and fungi. PCR/ESI-MS has also demonstrated the capacity to detect pathogenic organisms in clinical samples obtained following initiation of antimicrobial treatment[1]. Additional primer pairs target genes for antibiotic resistance or particular pathogenic features. Compared to clinical samples, the PCR/ESI-MS performs with 98.7% and 96.6% concordance at the genus and species levels, respectively[2].

PCR/ESI-MS (Ibis Biosciences, Carlsbad, CA) was performed on “culture negative” fluid and tissue samples from the patient: 2 mL of pleural fluid obtained at the time of initial chest tube insertion; and tissue from the loculated left sided empyema obtained during VATS. We followed the protocol previously described and validated by Kaleta *et al.*[2]. PCR/ESI-MS detected *mitis/viridans* group streptococci from the patient’s pleural fluid and tissue samples. As noted, the patient experienced complications associated with broad empiric antibiotic treatments that were likely attributable to clindamycin. Fortunately, the patient recovered completely from her infection, and the gastrointestinal side effects of antimicrobial treatment eventually resolved, but incorporation of molecular testing into her diagnostic evaluation may have averted the prolonged hospital stay.

*Mitis/viridans* streptococci are an uncommon cause of empyema. To determine if the patient was unique, IRB approval was obtained for retrospective review of all cases of pleural effusion in children ≤18 years of age hospitalized at our institution with empyema between January 1, 2013 and December 31, 2013. Patient consent was not needed for this retrospective chart review. We identified fourteen patients with parapneumonic effusion or empyema. Only three patients had evidence of septations and/or loculation in the effusion on chest imaging. Eight pleural fluid samples from six patients were submitted for culture: Gram stain and cultures were negative in 87.5% of specimens (7/8). All of the patients had been treated with antibiotics before pleural fluid was obtained for culture; the median duration of treatment was 8 days. The only positive pleural fluid culture grew methicillin susceptible *S. aureus* from fluid drained following two doses of antibiotic treatment, but when a second sample of pleural fluid was aspirated from this child after 4 days of antibiotic treatment, the cultures were negative.

## Results and discussion

Selecting appropriate antimicrobial therapy for patients with evidence of infection but negative cultures is a common dilemma especially in cases of empyema[3–5]. Pediatric Infectious Diseases Society and the Infectious Diseases Society of America guidelines for management of CAP recommend empiric treatment with IV ampicillin in fully immunized patients older than 5 years of age, but in a child with “culture negative” empyema there are no clear guidelines for treatment[6]. The patient was treated empirically with both ceftriaxone and clindamycin for both streptococcal and staphylococcal infection for two days before pleural fluid was sent for culture. This choice of antimicrobial therapy was influenced by the severity of her infection, including altered mental status demonstrated by listlessness, and CT scan findings of lung ischemia.

Even with widespread recognition that early intervention with VATS is associated with improved outcomes, the proportion of patients with negative pleural fluid and tissue cultures remains over 60% in patients with loculated effusions not responding to fibrinolysis[3–5]. In our retrospective review of all children ≤18 years of age hospitalized at our institution with parapneumonic effusion or empyema, 100% of the patients had been treated with antibiotics for at least 48 hours before pleural fluid was submitted for culture. We previously demonstrated bacterial pathogens are present and readily detected by molecular testing in 60% of patients with negative cultures when the samples have been obtained after 48 hours of empiric antibiotic treatment[1].

Molecular testing has improved sensitivity over culture for detection of bacterial pathogens from pleural fluid[3]. Unfortunately, molecular testing is not routinely available, and the turnaround time for send-out testing is too slow to impact routine care of inpatients. Specific PCR testing for MRSA is commonly available, but MRSA empyema and parapneumonic effusions remain extremely uncommon in pediatric patients[3, 5], and the applicability of PCR assays in clinical practice is limited by the positive vs. negative result inherent in conventional diagnostic PCR assays.

## Conclusions

Our case provides further support to the growing body of evidence that molecular diagnostics will play a role as an adjunct to conventional diagnostic microbiologic methods in culture negative cases of empyema and parapneumonic effusion. We suggest that when growth of organisms is inhibited by prior antibiotic treatment, particularly when Gram stain suggests non-viable organisms are present, molecular testing such as PCR/ESI-MS may provide an opportunity to narrow treatment. Our understanding of pleural space infections will undoubtedly benefit from more widespread use of molecular diagnostics such as nested PCR, 16S ribosomal gene sequencing, and PCR/ESI-MS. Future investigations conducted prospectively to analyze the diagnostic accuracy and utility of these assays should be conducted before empirical antimicrobial treatment is replaced by specific treatment targeting organisms identified in molecular assays.

### Key messages

1. Novel molecular diagnostics can have a significant impact on the diagnosis of serious culture negative infections of the lung and chest.
2. Understanding the role of *Streptococcus mitis/viridans* group in the etiology of empyema using advanced nucleic acid amplification technologies coupled with mass spectrometry can enlighten clinicians as to the impact of this pathogen in CAP and help with antibiotic stewardship.

## Abbreviations

PCR: Polymerase chain reaction
DNA: Deoxyribonucleic acid
PCR/ESI-MS: Polymerase chain reaction/electrospray ionization-mass spectrometry
VATS: Video-assisted thoracoscopy surgery.
